# Bio-removal of Heavy Metalsusing Iron-oxidizing Bacteria: A Novel Approach in Environmental Biotechnology

**DOI:** 10.22037/ijpr.2019.112474.13779

**Published:** 2020

**Authors:** Sareh Farahani, Abbas Akhavan Sepahi, Seyed Abbas Shojaosadati, Farzaneh Hosseini

**Affiliations:** a *Biology Department, Faculty of BioSciences, NorthTehran Branch, Islamic Azad University, Tehran, Iran.*; b *Chemical Engineering Department, Biotechnology Group, TarbiatModarres University, Tehran, Iran.*

**Keywords:** Heavy metals, Nickel, Mercury, Iron-oxidizing bacteria, Aquatic ecosystems, Bacillus sp

## Abstract

The pharmaceutical and hygienic productivity of wastewater containing pollutants, especially heavy metals such as nickel, andmercury are brought into the nature. Recently, bio-removal of heavy metals has attracted significant attention as an eco-friendly approach for the research departments of the pharmaceutical companies. In the current study, removal of heavy metals including mercury and nickel was assessed using isolatediron-oxidizing bacteria from different sources. To this end, bacterial populations were isolated from a variety of aquatic ecosystems; including Mahallat Pond, mountainous rivers, iron industry wastewater, and treated industrial wastewater. The bacteria were cultured and purified in iron-oxidizing media after which the removal of mercury and nickel was measured through culturing the isolated bacteria in 3 different media of Luria-Bertani, PHGII, and iron-oxidizing media containing the heavy metals (2 ppm). The results proved LB as a suitable medium for all the isolated bacteria in removing the heavy metals.It was shown that approximately 100% of the mercury was removed through the bacterial cultured in LB medium. The removal of nickel also reached its maximum of 30% by bacterial culture in LB medium. Then, the phylogenetic study according to 16S rDNA gene sequences showed thatthe isolated bacteria from iron industry wastewater was *Bacillus velezensis *CR-502 (T).In summary, this study demonstrated the impressive ability of these bacteria for mercury removal and theeffects of different mediaon the removal of mercury and nickel.

## Introduction

Due to the expansion of the pharmaceuticals and cosmetics industry, the construction of factories in these industries is also on the rise. Medicinal products in such factories are also contain waste materials which are harmful to the environment. Today, several hundred active pharmaceutical ingredients (APIs) have been found in sewage water, surface water, groundwater, soil, air, or biota in concentrations from sub-ng/L to more than µg/L. Thus far, there are several examples of APIs convincingly shown to cause effects on organisms in the environment (1). Iron-oxidizing bacteria are considered as one of the most important species in environmental biotechnology; since their significant both wreckingand beneficial effect on the environment and industrial facilities. Although these microorganisms are account for the formation of acid mine drainage, enhancing metal-surfacecorrosion, and water pipes clogging, their potentialsyn removing heavy metals have attracted a great deal of attention.Iron (Fe) is one of the most abundant elements on earth and a major component of the oceanic crust (2) whichis largely accumulated in the shape of banded iron formations (BIFs; oxidized deposits of Pre-Cambrian age) in the lithosphere (3) (approximately 28% w/w). Ehrenberg and Winogradsky, pioneer microbiologistsin the 19^th ^century, discoveredtheso-called “iron bacteria” that oxidize iron II (Fe^2+^, ferrous iron) to iron III (Fe^3+^, ferric iron) as biocatalysts (3). The significance of these bacteria in the biogeochemical cycling of iron has been broadly recognized over the past two decades (4). The ‘iron bacteria’ are a collection of morphologically and phylogenetically heterogeneous prokaryotes. While species of iron-oxidizing bacteria (IOB) can be found in many different phyla, most belong to the Proteobacteria.All the knowniron-oxidizing bacteria are oxygen-dependent, neutrophilic, and lithotrophic (5). Iron-oxidizing bacteria are capable of oxidizing ferrous iron abiotically in waters rich in oxygen and neutral acidity. The interface between aerobic and anoxic areas in sediments and ground watersare usually colonizedby aerobic, neutrophilic iron oxidizers, which are called ‘gradient’ organisms. Iron-oxidizing bacteria are majorlyinvolved in biogeochemical cycles, microbial corrosion, and removal of heavy metals (6). According to Fergusson (1990), heavy metals are highly densemetallic elements compared to watermolecules (7). It is assumed that there is an association between heaviness and toxicity of metals (8) namely heavy metals are ofhigh toxicity even at low-levelexposure.Recently, warnings have been issued regarding the ecological and global public health risks associated with the environmental contamination brought about by these metals.In addition, because of the growing usage of heavy metals in industrial, agricultural, domestic, and technological applications, theirexposure rate increasessignificantly (9). However, there is a lack of toxicity studies for various groups of chemicals, for example: pharmaceuticals ,nano-particles, and industrial chemicals. This has resulted in difficulties when performing risk assessments since any risk assessment relies on the availability of reliable and relevant studies. Pharmaceuticals were first identified to pose environmental risks in the 1990s, and since then the number of available monitoring and effect studies has increased steadily.

To name a few, mercury, nickel, arsenic, barium, chromium, lead, selenium, and silverarenaturally occurring heavy metals that are found in different environments in trace concentrations. However, their presence and exposure in higher concentrations can impact human health and must be cautioned (8).

Mercury, as one of the most dangerous heavy metals, may combine with other elements and form organic and inorganic compounds (8, 10). The three forms of mercury (elemental form, and organic/inorganic compounds) have showndifferent toxicity features (11). Various chemical forms of mercury including elemental mercury vapor (Hg^0^), inorganic mercurous (Hg^+1^), mercuric (Hg^+2^), and the organic mercury compounds exist in nature and affect both human and animal health 12).Severe alterations in the body tissues are caused by mercury that bring about negative health effects (13). Permanent damages to brain, kidney, and fetus are very likely when individuals expose to high levels of mercury (14). Short-term exposure to considerable amounts of mercury vapors can cause various damages to lung and skin (14). Moreover, its presence in soil and water causes microorganisms to convert them to abioaccumulating toxin that brings about important health issues such as human carcinogenesis (14). Therefore, to prevent these damages, the maximum allowed mercury in drinkable water and sea food are 2 and 1 ppm, respectively (14).

Another carcinogenic metal thatcauses environmental pollution is nickel.Warnings have been issued by New York University, at School of Medicine, regarding the relationship between chronic exposure to this metal and the increased risk of lung cancer, cardiovascular diseases, neurological deficits, the developmental deficit in childhood, and high blood pressure (15). Free radicals generated due to tonickel exposure causekidneys, liver, andoxidative damage (16). Researchers at the Dominican University of California believe that nickel exposure and breast cancer are closely correlated (17). Moreover, studies have shown that nickeltoxicity damages the reproductive system significantly that leadstoinfertility, miscarriage, birth defects, and nervous system defects (18, 19).

In this study, bacterial populations were isolated from a variety of aquatic ecosystems; including Mahallat Pond, mountainous rivers, activated sludge, iron industry wastewater, and treated industrial wastewater after which they were cultivated and purified in iron-oxidizing media. Then, the purified bacteria were culturedin different media having 2 ppm of mercury and nickel to assess their ability to remove these heavy metals. 

## Experimental

*Materials*

All the materials were purchased from Merckcompany (Germany) andused as provided.

*Samples acquisition*

Samples of muddy waterwere acquiredfrom a variety of aquatic ecosystems,including pond, rivers, activated sludge, and industrial wastewaters.Thesamples were taken from 10 to 20 cm depth and 500 mL volume. All sampled containers were sterilized.The samples were quickly transferred to the laboratory and stored at 4 °C until cultivation.

*Media preparation*

The employed modified iron-oxidizing medium in this study consisted of 3.0 g (NH_4_)_2_SO_4_, 0.5 g K_2_HPO_4_, 0.5gMgSO_4_·7H_2_O, 0.1 g KCl, 0.01gCa (NO_3_)_2_ and 300.0mL FeSO_4_·7H_2_O solution. Composition per 300.0mLIron sulphate solution add 44.22 g FeSO_4_·7H_2_O (20).PHG II medium contained 4.0 g/L peptone, 2.0 g/L glucose, 1.0 g/L yeast extract. To make solid media, 15 g/L agar was added to the as-prepared liquid media. After preparing the all media and adjusting their acidity to 6.5-7, they were autoclaved for 15 min 121 °C for sterilization.

*Microbial inoculation and cultivation*

First, the iron-oxidizing media (100 mL) was inoculated with water samples (10 mL) separately. They were cultured for 2 weeks at 25 °C in an incubator shaker (KS 4000i control, IKA, Germany) with a shaking speed of 160 rpm (6). After observing considerable turbidity, they were transferred to the prepared solid medium in platesfor further tests. To obtain the growth curve for the isolated bacteria, they were cultured in LB medium containing yeast extract 5.0 g/L, peptone from casein 10.0 g/L and NaCl 10.0 g/L. 

*Bacterial growth rate evaluation*

To investigate the growth rate, the bacteria were first passaged in solid culture medium and then one colony was inoculated into LB broth medium. The ratio of culture medium to bacteria is 9:1. The culture media containing bacteria were monitored daily for 10 days using spectrophotometer (PG Instruments) with wavelength 600 nm. Finally,the growth curve was plotted for 5 bacteria.

*Removalof heavy metals*

To investigate the removal of heavy metals, the isolated bacteria were cultured separately in 10 mL of LB, PHG II, and iron-oxidizing media in whichever there was 2 ppm of mercury and nickelin form of HgCl_2_ and NiCl_2_.6H_2_O. This step was accomplished in incubator shaker (150 rpm) at 28 °C for 72 h.

*Detection of ferric ions in iron oxidizing bacteria*

The presence of ferric ions in bacteria were detected using Potassium ferrocyanide (K_3_Fe(CN)_6_). Initially, an aliqout of bacteria was spread on a 0.45μm filter membrane and allowed to dry. The membrane was placed on a paper filter soaked with a 5% solution of potassium ferrocyanide. This was allowed to incubate for 10 min. Then, a new paper filter was soaked with 5% HCl and the membrane was laid inside it. Potassium blue precipitate was produced. HCl induced the solubilization of Fe^3+^.Finally, the membrane was dried and observed with light microscope.

*Microscopy*

Prior to imaging the selected sample using scanning electron microscopy (SEM, ZEISS, SIGMA VP-500), it was freeze-dried (Christ, α2-4LFCPlus) for 24 h. The sample was imaged at an accelerating voltage of 10 kV after sputter-coating with gold.

*Nigrosin stainin*g

Nigrosin as an acidic stain was used for negative staining of the bacteria. To this end, one colony of the bacteria was transferred on a glass slide and was mixed with 2 droplets of the stain. After complete mixing and forming a film, the cells were investigated using a light microscope (Nikon Alphaphot-2 YS2) (21).

*Phylogenetic study*

To study the selected species phylogenetically, colonies of the selected bacteria were used for genomic DNA extraction and further PCR amplification of 16S rDNA genes. To this end, the universal primers which were used for the amplification of 16S rDNA were 27F (5′-AGAGTTTGATCCTGGCTCAG-3′) and 1492R (5′-CGGCTACCTTGTTACGACTT-3′) (22) and the amplified DNA was sequenced through 3730/3730 x lDNA Analyzers (Thermo Fisher Scientific). Using BLAST and based on the homology in the Genbank DNA databases, the species were identified. Finally, the phylogenetic tree was illustrated using the Mega 7 software.

## Results

*Characteristics of sampling areas*.

*Isolation and cultural characteristics*

The liquid media were dark-red, after incubation for 2 weeks. Five types of colonies were grown on solid media. Their morphology was white, powdery and strictly sticking to the medium. Colonies were spread on fresh iron solid media because filamentous bacteria may contain Fe (II)-oxidizing bacteria Liu *et al*. (2013) (23).

*Growth curve*

**Figure 1:**
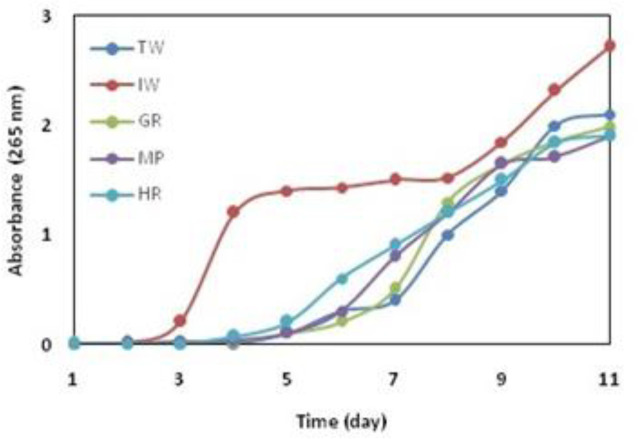
Growth of the selected colonies in LB medium for 11 days

The growth curve was checked for five selected colonies (Figure.1). Bacteria were grown in Luria-Bertani (LB) broth for 10 days with each showing different turbidity. Most of the changes were in two samples including refined industrial sewage and pond. The bacteria isolated from the iron industry wastewater were more turbidity in the LB medium than other bacteria. It can be concluded that bacteria were facultative chemoorganotroph (3). 

*Ferric presence in bacteria*

During the reaction, each of the five samples with potassium ferrocyanide was dark-blue in color indicating the presence of ferric ions in isolated bacteria (23).

*Microscopy*

**Figure 2:**
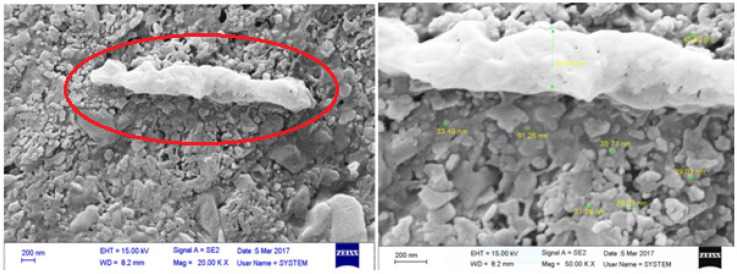
The scanning electron microscope micrographs from the bacteria isolated from iron industry wastewater

**Figure 3:**
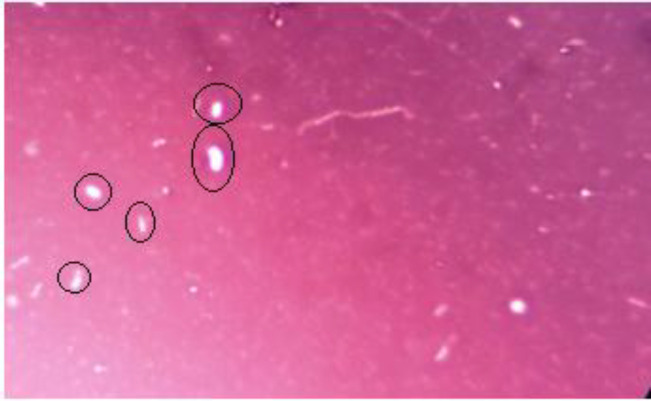
The bacteria images after nigrosin staining (inside the rings) x 1000

Iron-oxidizing bacteria are often filamentous or bacilli. In this study, the form of these bacteria was rod shape from ironindustry wastewater (Figure 2). Furthermore, due to the presence of nanoparticles, it can be concluded that the bacteria have the ability to synthesize nanoparticles (Figure 2). A preliminary study of iron bacteria using negative staining method was nigrosin (Figure 3). 

*Removal of heavy metals*

**Figure 4:**
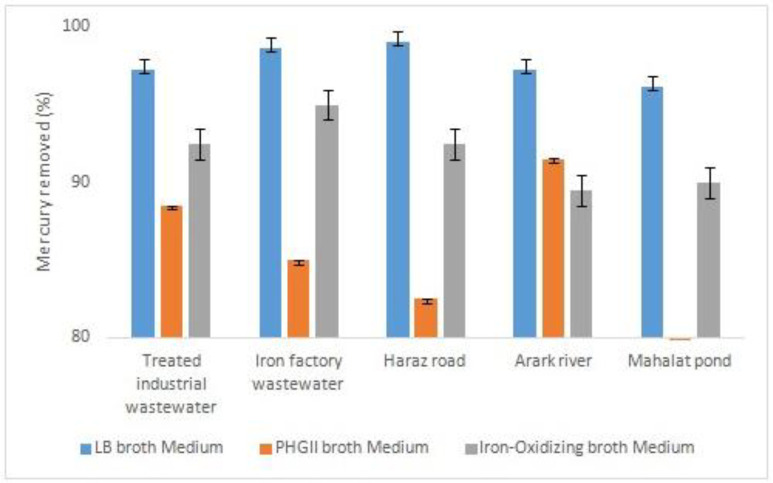
The result of atomic absorption in mercury removal

**Figure 5:**
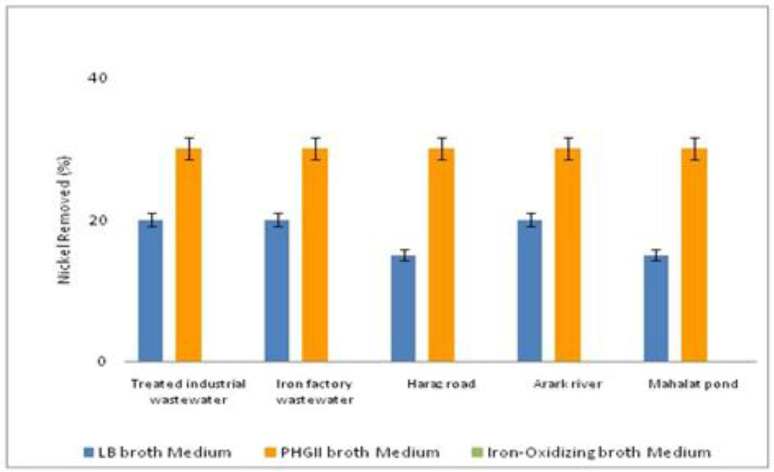
The result of atomic absorption in nickel removal

To assess the bacteria capability to remove heavy metals, they were cultured in 3 different media with 2 ppm of nickel and mercury. As depicted in Figure 4, the five bacteria samples removed all the mercury element when they were cultured in LB broth medium. It was about the same when iron-oxidizing broth medium was used for culture. However, bacterial culture in PHG II broth medium decreased the mercury removal, specifically in MP bacteria sample. When compared to nickel removal, the bacteria samples cultured in iron-oxidizing broth medium was not able to remove any nickel (Figure 5). Nevertheless, PHG II broth medium caused all the bacteria samples to remove nickel up to 30%. Thus, it is proved PHG II broth medium as a more suitable bacteria culture for removal of heavy metals.

*16S rDNA nucleotides sequence accession numbers*

The results of sequencing of 16S rDNA gene from the iron industry wastewater, showed that the strain of 1511 nucleotides with 99.7% of the strain of *Bacillus velezensis *CR-502 (T) has a phylogenic affinity access number AY603658.

**Figure 6:**
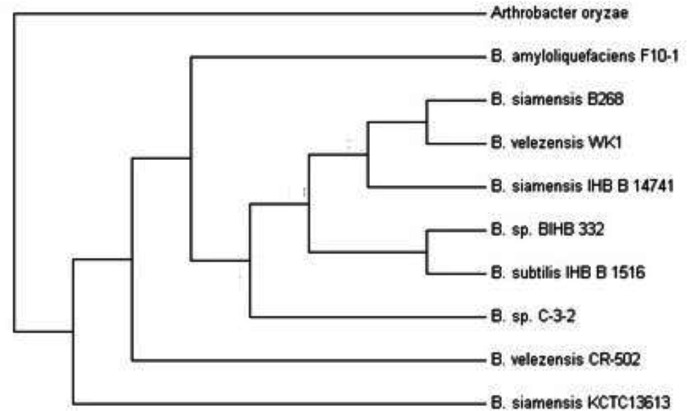
Neighbor-joining phylogenetic tree (500 bootstraps of 16S rRNA) for *Bacillus velezensis *CR-502 (T) isolated from iron industry wastewater

The phylogenetic tree that wasdrawn using the Mega 7 software, refers to *Bacillus velezensis *CR-502 (T) (Figure 6).

**Table.1:** The samples collected from several regions of Iran

**Temperature (°C)**	**pH**	**Geographical coordinates**	**Date**	**Source**
20	7	51°28′35″N, 35°49′13″E	2017.05.08	Activated sludge^1^(AS)
10	6	49°41′26″N, 34°41′9″E	2017.04.05	Gerdooriver^2^ (GR)
9	8	52°31′58″N, 13°23′8″E	2017.02.21	Rivers along Haraz road (HR)
12	7	34° 8′ 22″ N, 50° 3′ 35″ E	2017.05.04	Iron industry wastewater (IW)
12	7	33°58′44″N, 58°33′36″E	2017.04.20	Mahallat pond^3^ (MP)
20	7	50°13′7″N, 34°59′13″E	2017.05.03	Treated industrial wastewater (TW)

^1^Taken from Sahebgharanieh wastewater treatment plant

^2^A seasonal river located in Arak province

^3^ Isfahan province

## Discussion

In the present study the bioremediated bacteria were isolated from Pond, mountainous rivers, iron industry wastewater, and treated industrial wastewater, while in other researches, the microbes were isolated from soil landfarming and landspreading (23) polluted river sediments (24) sewage sludge (25) industrial effluents (26) sewage (27) aqueous solutions (28) anaerobically digested sewage sludge (29) and contaminated soils (30). An increase in emission of heavy metals has been always announced asan important health alert for human beings. On the other hand, the great and also required progress in different industrial sectors increases the emission of heavy metals inevitably. Among many methods suggested for removal of heavy metals from the environment, bio-based approaches have been always introduced as efficient, inexpensive, and eco-friendly techniques for reaching this end.In this study, iron-oxidizing bacteria isolated from different aquatic sources have been employed as living agents for removal of heavy metals. The isolation was performed using iron-reducing media as a common method (6, 32). Since the isolated bacteria were able to grow in organic and non-organic media, they are probably facultative chemoorganotrophs (33). Based on the phylogenetic studies, these bacteria are commonly related to neutrophilic lithotrophic proteobacteria (5). However, the bacteria isolated in this study showed different characteristics similar to the study accomplished by Liu *et al. *(34).The SEM micrographs confirmed their rod-shaped morphology that is one of the most common morphologies of iron-reducing bacteria (34, 35). The isolated strains from IW were identified as *Bacillus velezensis *CR-502 (T), which was from *Bacillus* genus. In a study, the isolated bacteria from calcareous soils were grown in the iron-oxidizing medium and were able to reduce Fe^3+^ to Fe^2+^. Similarly, these bacteria were proved to be from *Bacillus* genus (36).

The desired bacteria can be isolated from different sources including various water resources, soil, mine areas, and different wastewaters (32, 34, 36-37). In this study, the bacteria were isolated from different aquatic environments including rivers, treated industrial wastewater,iron industry wastewater, and a pond while it could not be isolated from activated sludge. These species showed good capability in the removal of heavy metals from the media contaminated with mercury and nickel. The efficiency of mercury removal was at the highest (more than 95%) when the bacteria were cultured in LB broth medium. However, this decreased to between 60-90% depending on the bacteria sources when using PHG II broth medium. In contrast, nickel was removed with a maximum of about 30 % in PHG II medium.

Technically speaking, living bacteria in a specific environment have been adapted to its physical and chemical conditions (35). Giovanella *et al*. (2016), isolated mercury resistant bacteria and identified *Pseudomonas *sp. B50A. Mercuric (II) reductasewasproduced by this bacteria. *Pseudomonas sp. *B50 Aremoved 86% of the mercury (1). But, in the present study mercury removed 100% by *Bacillus velezensis* from industerial waste water. Besides,Kailasam *et al*. (2017), investigated removal of mercury by *Vibrio fluvialis* from industrial effluents and their reported mercury has been removed 60% (4), while in the present research it removed 100% by *Bacillus* sp. Remarkably, bacteria can be multi-metal tolerant or resistant. Sunil *et al*. (2015), isolated *Streptomycesflavomacrosporus* from paddy field irrigated with industrial effluents. This bacterium had the ability to bioremediation a few metals (9). Also in the present study, *Bacillus* sp. removed mercry and nickel about 100% and 30%, respectively.

Mercury resistant bacteria by removing mercuric ions to metallic mercury, remove mercury from the environments. In a study, *Escherichia coli (E. coli.) *KP245 reduced the 70 mg/L mercury in the sewage to 2.5 mg/L over two weeks (5). In a similar study, *E*.*coli *and *B. subtilis* removed heavy metals (6). Moreover, Xiang *et al.* (2000), used indigenous iron-oxidizing bacteria isolated from anaerobically digested sewage sludge for removal of heavy metals.They, removed the heavy metals including nickel from the sweage sludge with the yeild of 54.4% (7) while in the present study, nickel was removed with the yield of 30%. Likewise, in a study, the heavy metals were removed in contaminated soils by iron oxidizing bacteria. In addition, heavy metal depletion lasted from 18–30 days to 2–8 days (8). 

## Conclusion

In this study, a simple and ecofriendly method was evaluated for removal of nickel and mercury from different media. Significantly, 100% of mercury was removed through the bacterial cultured in LB medium, while 30% of nickel was removed in the same condition. Therefore, the bacteria isolated from a polluted environment can be well optimized to reduce the present contaminations. Since these bacteria are capable of oxidizingiron or other heavy metals, they can be employed in microbial fuel cells and microbial electrolysis cells as novel bioelectrochemical systems in which microorganisms produce electricity while treating different wastewaters.Futhermore, this economic and ecofriendly approach can be successfully used to remove heavy metals from the wastewater of pharmaceutical companies. 
